# Performance of early risk assessment tools to predict the later development of gestational diabetes

**DOI:** 10.1111/eci.13630

**Published:** 2021-06-18

**Authors:** Grammata Kotzaeridi, Julia Blätter, Daniel Eppel, Ingo Rosicky, Martina Mittlböck, Gülen Yerlikaya‐Schatten, Christian Schatten, Peter Husslein, Wolfgang Eppel, Evelyn A. Huhn, Andrea Tura, Christian S. Göbl

**Affiliations:** ^1^ Department of Obstetrics and Gynaecology Medical University of Vienna Vienna Austria; ^2^ Center of Medical Statistics, Informatics, and Intelligent Systems Section for Clinical Biometrics Medical University of Vienna Vienna Austria; ^3^ Department of Obstetrics and Gynaecology University Hospital Basel Basel Switzerland; ^4^ Metabolic Unit CNR Institute of Neuroscience Padova Italy

**Keywords:** early gestation, gestational diabetes mellitus, risk prediction model

## Abstract

**Background:**

Several prognostic models for gestational diabetes mellitus (GDM) are provided in the literature; however, their clinical significance has not been thoroughly evaluated, especially with regard to application at early gestation and in accordance with the most recent diagnostic criteria. This external validation study aimed to assess the predictive accuracy of published risk estimation models for the later development of GDM at early pregnancy.

**Methods:**

In this cohort study, we prospectively included 1132 pregnant women. Risk evaluation was performed before 16 + 0 weeks of gestation including a routine laboratory examination. Study participants were followed‐up until delivery to assess GDM status according to the IADPSG 2010 diagnostic criteria. Fifteen clinical prediction models were calculated according to the published literature.

**Results:**

Gestational diabetes mellitus was diagnosed in 239 women, that is 21.1% of the study participants. Discrimination was assessed by the area under the ROC curve and ranged between 60.7% and 76.9%, corresponding to an acceptable accuracy. With some exceptions, calibration performance was poor as most models were developed based on older diagnostic criteria with lower prevalence and therefore tended to underestimate the risk of GDM. The highest variable importance scores were observed for history of GDM and routine laboratory parameters.

**Conclusions:**

Most prediction models showed acceptable accuracy in terms of discrimination but lacked in calibration, which was strongly dependent on study settings. Simple biochemical variables such as fasting glucose, HbA1c and triglycerides can improve risk prediction. One model consisting of clinical and laboratory parameters showed satisfactory accuracy and could be used for further investigations.

## 1 INTRODUCTION

According to the most recent WHO recommendations, gestational diabetes mellitus (GDM) is diagnosed in the late second or early third trimester by using a 75 g oral glucose tolerance test (OGTT).^1^ Although there is no consensus about screening algorithms and diagnostic criteria for GDM before 24 weeks of gestation, risk stratification at early pregnancy could be beneficial to reduce diabetes associated co‐morbidities due to timely interventions such as physical exercise and dietary changes.^2^ For this purpose, anamnestic risk factors (maternal age, overweight or obesity, ethnicity, history of GDM in previous pregnancy and others) can be used to distinguish women with low and high risk for the later development of hyperglycaemia.^3^ Although risk factor‐based screening is criticized to have limited diagnostic accuracy, it has the advantage of cost‐effectiveness and simplicity as clinical risk factors are easily obtainable from the patient's history already at early gestation.^4^ Recently, some authors suggested that risk factor‐based screening could be considerably improved by use of clinical prediction models consisting of statistical combinations of several GDM associated risk factors.^5^ Their predictive performance could possibly be further improved by inclusion of fasting plasma glucose and other laboratory measurements such as triglycerides or glycated haemoglobin A1c (HbA1c).^6^ As a consequence, various risk estimation tools have been published in the previous years. However, most of these previous studies used different GDM diagnosis criteria; therefore, their clinical benefit when applied to the most recent GDM definition is not well investigated yet.

This study aims to assess the performance of published low‐invasive prediction models (ie risk estimation containing anamnestic data and routinely available fasting laboratory measurements) to predict the later development of GDM as defined by the recent WHO recommendations^1^ as primary objective. Discrimination and calibration of several models, as well as the importance of included variables, were assessed in an independent population, and their possible clinical benefit was investigated and discussed.

## 2 METHODS

### 2.1 Study design and participants

In this prospective cohort study, we included 1132 pregnant women. A flow chart with detailed information about included and excluded patients is provided in the supplemental material (Figure S1). Study participants (≥18 years) were consecutively recruited among pregnant women attending our outpatient clinic (Department of Obstetrics and Gynaecology, Division of Obstetrics and Feto‐Maternal Medicine, Medical University of Vienna) between January 2016 and July 2019. Women with pre‐existing diabetes as well as women with history of bariatric surgery were excluded. A broad risk evaluation was performed before 16 + 0 weeks of gestation, including the assessment of pre‐gestational body mass index (BMI, based on the self‐reported pre‐gestational weight), BMI at initial contact, maternal age, parity, obstetric history, family history of diabetes (first and/or second degree) and history of GDM in previous pregnancies. A detailed assessment of ethnicity was required for some prediction models and included the following ethnic categories: Caucasian (n = 881, 77.8%), African/Black (n = 47, 4.2%), Middle Eastern and North African (n = 76, 6.7%), Asian (n = 118, 10.4%), Latin American (n = 9, 0.8%) and Caribbean (n = 1, 0.1%). Due to reported differences in GDM risk between different Asian ethnic groups, the ethnic category ‘Asian’ was further subdivided into the subcategories South Asian (n = 82, 7.2%), Southeast Asian (n = 18, 1.6%), East Asian (n = 11, 1.0%) and Central Asian (n = 7, 0.6%). Obstetric history included adverse obstetric outcomes such as unexplained foetal death at >20 weeks of gestation, spontaneous abortions and foetal anomalies despite normal karyotype. To match the definitions used in certain prediction models, macrosomia in previous gestation was defined as birthweight greater than 4000 g, whereas large for gestational age (LGA) was defined as birthweight greater than 90th percentile using INTERGROWTH‐21st newborn growth standards, which were also used to determine the birth weight z‐scores.^7^ In addition, a routine laboratory examination was performed at baseline visit with assessments of plasma glucose, triglycerides, glycated haemoglobin A1c (HbA1c) and urine glucose after at least 8 hours of fasting. Participants with fasting plasma glucose or HbA1c equal or exceeding 126 mg/dL or 6.5%, respectively, were excluded from the cohort. Thereafter, participants were followed‐up until delivery to assess glycaemic status by using a 75 g 2 h OGTT according to the International Diabetes in Pregnancy Study Groups (IADPSG) recommendations^8^ in a universal screening. In cases where fasting glucose was ≥92 mg/dL before 24 weeks of gestation, presence of GDM was verified by either early OGTT (n = 33) or self‐monitored blood glucose (n = 25) in accordance with our national guidelines.^9^ The majority of those patients had a history of GDM in previous pregnancies and showed elevated blood glucose levels throughout their entire current pregnancy, often requiring pharmacological intervention with insulin and/or metformin. Women with negative OGTT results, who received glucose lowering medication during follow‐up due to macrosomia and after confirmation of hyperglycaemia through self‐monitoring of blood glucose, were also classified as GDM (n = 11). All laboratory parameters were measured according to the standard laboratory methods at our certified Department of Medical and Chemical Laboratory Diagnostics (http://www.kimcl.at). Plasma glucose concentrations were measured by the hexokinase method with a coefficient of variation (CV) of 1.3%. HbA1c was assessed by high performance liquid chromatography, IFCC standardized and DCCT aligned (CV=1.8%). Fifteen clinical prediction models were identified and calculated according to the published literature after performing systematic searches in PubMed, including five ‘sum score models’,^10^, ^11^, ^12^, ^13^, ^14^ nine ‘propensity score models’^5^, ^6^, ^15^, ^16^, ^17^, ^18^, ^19^, ^20^ and one decision tree model.^21^ A mathematical description of the prediction models is provided in the supplemental material (Table S1). Three additional models were identified but not included in this study as they either used a simple combination of body weight / BMI and maternal age^22^, ^23^ or included predictors or biomarkers which are not available in clinical routine, such as tissue plasminogen activator inhibitor or adipokines.^24^ Moreover, the full prediction rule was not provided in two of these studies.^22^, ^24^

The study was approved by the Ethics Committee of the Medical University of Vienna (protocol number 1937/2015) and performed in accordance with the Declaration of Helsinki. Written informed consent was obtained from all participants. Reporting of the study conforms to the broad EQUATOR guidelines.^25^

### 2.2 Statistical analysis

For statistical analysis, we performed complete case analysis. Women with known glycaemic status in late pregnancy but with variables missing were still included in the analysis. Prediction models containing the missing variables were not calculated for those women.

Continuous variables were summarized by mean ±standard deviation or as median and interquartile ranges (IQR) where appropriate and compared by Welch's *t* test or the Wilcoxon rank sum test, respectively. Categorical variables were summarized by counts and percentages and compared by Pearson's chi‐squared test. Discrimination (ie the ability of a model to separate pregnant women with disease from those without disease) was assessed by receiver operating characteristics curve (ROC) analysis. Calibration (ie the agreement between predicted vs. observed probability of having the disease) was assessed for propensity score models before and after recalibration by use of calibration plots.^26^ In addition, we used decision curve analysis to assess the net benefit of the four prediction models showing the best discrimination as compared to default strategies (ie treating all patients or no patients as having the disease).^27^ The net benefit is expressed as the net proportion of true positive cases: net benefit = true positive / *n* – false positive / *n* × *w* where *n* is the total number of cases and *w* is a relative weight equal to the odds of a threshold probability. A net benefit of 0.2 means that 20% of the study participants would be correctly classified as true positive cases at a respective cut‐off probability of a model.^26^ Finally, random decision forests with ntree  =  10^4^ were created by the conditional inference framework (cforest) to derive measures of variable importance. Variable importance is calculated as the average difference in predictive accuracy before and after random permutation of the values of a predictor variable over all (ie ten thousand) trees.^28^, ^29^ Statistical analysis was performed with R (version 3.5.3) and contributing packages (especially ‘pROC’, ‘rms’, ‘party’ and ‘rmda’).^30^ With a sample size of 1132 patients (239 cases with GDM), we are able to identify an area under the ROC of at least 57.4% compared to the noninformative ROC‐AUC value of 50% with a power of 95% and a two‐sided α‐error probability of 5%. When the total sample size is 1132, where group 1 has a sample size of 239 and group 2 has a sample size of 893, a two‐sided 95% confidence level with an area under the ROC equal to 0.65/0.7/0.75 produces a confidence interval width of 0.041/0.040/0.038. A two‐sided *P*‐value of ≤.05 was considered statistically significant.

## 3 RESULTS

### 3.1 Characteristics of the study cohort

Among a total of 1,132 women included in this study, n = 239 (21.1%) developed GDM, whereas n = 893 (78.9%) remained normal glucose tolerant and did not develop GDM (NGT). A comparison of baseline characteristics of both groups is provided in Table 1. Patients who developed hyperglycaemia were older, had significantly higher BMI, body weight and blood pressure already at the beginning of pregnancy and were characterized by higher HbA1c, fasting plasma glucose and triglyceride concentrations. History of GDM in previous pregnancies as well as family history of type 2 diabetes and non‐Caucasian ethnicity were more often observed in women who developed GDM. Moreover, history of LGA delivery was more often observed in women who developed GDM.

**TABLE 1:** Characteristics of the study sample at study entry

	n	NGT (n = 893)	n	GDM (n = 239)	*P*‐value
Age (years)	893	31.4 ± 5.8	239	32.8 ± 5.7	<.001
Parity (≥1)	893	541 (60.6)	239	165 (69.0)	.017
GDM in previous pregnancies	893	52 (5.8)	239	68 (28.5)	<.001
Ethnicity (non‐Caucasian)	893	184 (20.6)	239	67 (28.0)	.014
Height (cm)	893	165 ± 6.8	239	164 ± 6.8	.008
Weight, before pregnancy (kg)	893	66.1 ± 14.7	239	72.5 ± 16.3	<.001
Weight, current (kg)	893	67.5 ± 14.6	239	74.7 ± 16.4	<.001
BMI, before pregnancy (kg/m^2^)	893	24.3 ± 5.2	239	27.1 ± 5.7	<.001
BMI, current (kg/m^2^)	893	24.8 ± 5.1	239	27.9 ± 5.7	<.001
Family history (1st degree)	893	214 (24.0)	239	89 (37.2)	<.001
Family history (1st and 2nd degree)	893	386 (43.2)	239	143 (59.8)	<.001
Glucosuria (>40 mg/dL)	893	9 (1.0)	239	3 (1.3)	.740
RRS (mmHG)	892	118 ± 12.8	239	121 ± 11.8	.005
RRD (mmHG)	892	76 ± 10.0	239	79 ± 9.5	<.001
Prior LGA delivery (>90 perc)	893	84 (9.4)	239	38 (15.9)	.004
Prior macrosomia (>4000 g)	893	55 (6.2)	239	27 (11.3)	.006
Preconception dyslipidaemia	891	18 (2.0)	238	7 (2.9)	.391
Assisted reproduction	893	87 (9.7)	239	35 (14.6)	.030
Multiple pregnancy	893	107 (12.0)	239	21 (8.8)	.166
Smoking status	893	352 (39.4)	239	86 (36.0)	.333
FPG (mg/dL)	848	80.6 ± 5.8	224	86.0 ± 7.8	<.001
Triglycerides (mg/dL)	848	107 (82‐139)	225	130 (99‐165)	<.001
HbA1c (%)	857	4.95 ± 0.29	227	5.13 ± 0.30	<.001

Note

Data are mean ± SD or median (IQR) and count (%) for women remaining normal glucose tolerant (NGT) vs. patients developing gestational diabetes (GDM).

Abbreviations: BMI, body mass index; FPG, fasting plasma glucose; HbA1c, glycated haemoglobin A1c; LGA, large for gestational age neonates; RRD, diastolic blood pressure; RRS, systolic blood pressure.

### 3.2 Discrimination of the investigated prediction models

A brief summary of the prediction models analysed in this study including predictor variables and measures of discrimination is presented in Table 2. The areas under the ROC curve (ROC‐AUC) ranged between 60.7% and 76.9%, corresponding to a moderate to fair accuracy. In general, ‘propensity score’ models (ie prognostic models that calculated the probability for GDM on a continuous scale)^5^, ^6^, ^15^, ^16^, ^17^, ^18^, ^19^, ^20^ showed improved predictive performance as compared to ‘sum score’ models^10^, ^11^, ^12^, ^13^, ^14^ and the ‘decision tree’ model,^21^ whereby especially ‘propensity score’ models showed improved discrimination as compared to maternal age (ROC‐AUC: 56.6%, 95%CI: 52.6‐60.7), pre‐gestational BMI (ROC‐AUC: 66.0%, 95%CI: 62.1‐69.9) or BMI at study entry (ROC‐AUC: 67.5%, 95%CI: 63.7‐71.3) alone. The results remained comparable in a sensitivity analysis after excluding mothers with multiple pregnancies (n = 128), although discrimination tended to be lower in another sensitivity analysis including only nulliparous women (n = 426), with ROC‐AUC statistics ranging between 56.7% and 73.9% (supplemental material Table S2).

**TABLE 2:** Summary and discrimination performance of clinical risk prediction models evaluated in this study ordered by type of the model and publication year

Author	Included variables	ROC‐AUC (%)	95% CI
Naylor 1997^10^	Sum score model: Age; Pre‐pregnancy BMI; Ethnic origin	65.5	61.7‐69.2
Caliskan 2004^11^	Sum score model: Age; Pre‐pregnancy BMI; Prior adverse obstetric outcome; Family history of diabetes; Prior macrosomia	64.5	60.8‐68.3
Shirazian 2009^14^	Sum score model: Αge; Pre‐pregnancy BMI; Family history of diabetes	60.7	56.8‐64.7
Phaloprakarn 2009^13^	Sum score model: Age; First trimester BMI; Family history of diabetes; Prior macrosomia; History of ≥2 abortions	67.6	63.9‐71.3
Teede 2011^12^	Sum score model: Age; First trimester BMI; Ethnic origin; Family history of diabetes; History of GDM	68.9	65.2‐72.7
Pintaudi 2014^21^	Decision tree model: FPG; pre‐pregnancy BMI	67.7	63.8‐71.6
van Leeuwen 2010^5^	Propensity score model: Ethnic origin; Family history of diabetes; Multiparity; Pre‐pregnancy BMI	70.8	67.1‐74.5
Nanda 2011^15^	Propensity score model: Age; First trimester BMI; Ethnic origin; History of GDM; Prior macrosomia	72.9	69.1‐76.6
Göbl 2012^17^	Propensity score model: History of GDM; Glycosuria; Age; Family history of diabetes; Preconception dyslipidaemia; Ethnic origin; FPG	71.7	67.7‐75.6
Savona‐Ventura 2013^20^	Propensity score model: FPG; Age; Diastolic blood pressure	65.2	61.0‐69.4
Syngelaki 2015^16^	Propensity score model: History of GDM; First trimester weight; Parity; Age; Height; Family history; Ovulation drugs; Ethnic origin; Birth weight	71.5	67.7‐75.3
Gabbay‐Benziv 2015^19^	Propensity score model: Age; Ethnic origin; History of GDM; Systolic blood pressure; First trimester BMI	71.6	67.9‐75.3
Sweeting 2017^18^	Propensity score model: History of GDM; Ethnic origin; Family history of diabetes; Parity; Αge; First trimester BMI	71.2	67.5‐74.9
Benhalima‐1 2020^6^	Propensity score model: Family history of diabetes; History of smoking before pregnancy; Ethnic origin; Age; Height; First trimester BMI; History of GDM	71.7	68.0‐75.4
Benhalima‐2 2020^6^	Propensity score model: History of GDM; FPG; Height; Triglycerides; Age; Ethnic origin; First trimester weight; Family history of diabetes; HbA1c	76.9	73.2‐80.6

Abbreviations: ROC‐AUC, area under the receiver operating characteristic curve; FPG, fasting plasma glucose; BMI, body mass index; HbA1c, glycated haemoglobin A1c.

### 3.3 Calibration of the investigated prediction models

**FIGURE 1:**
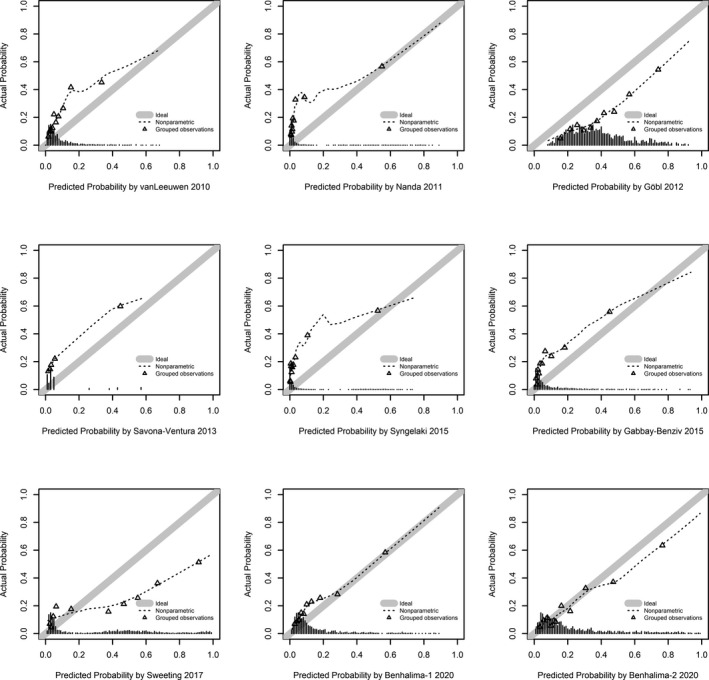
Calibration plots of prognostic models (calculated as they were originally published). Good calibration is observed if the dashed calibration line of the model is closely following the ideal calibration line (with an intercept of 0 and a slope of 1) as underlined with grey colour

**FIGURE 2:**
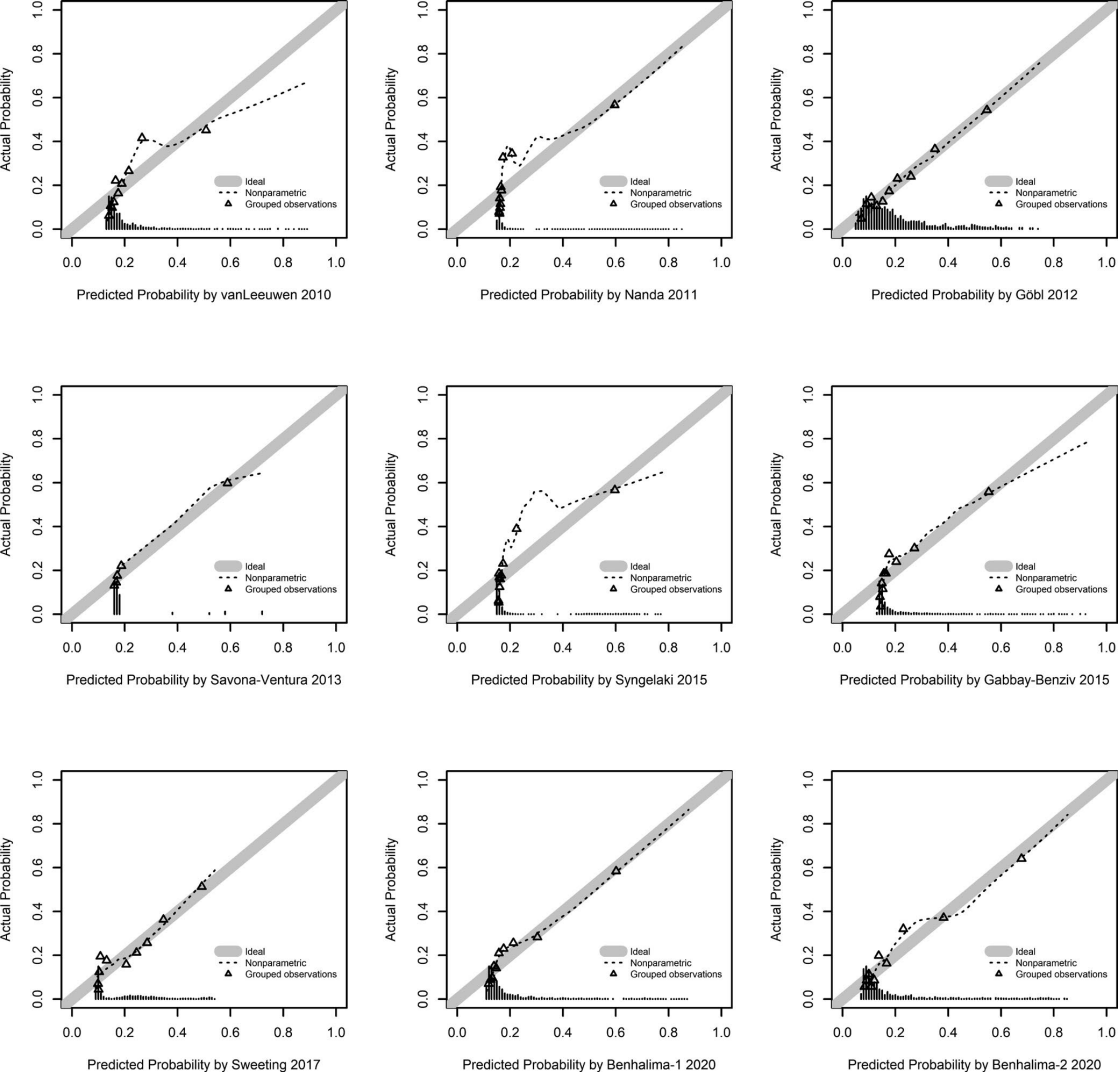
Calibration plots of prognostic models (after recalibration). Good calibration is observed if the dashed calibration line of the model is closely following the ideal calibration line (with an intercept of 0 and a slope of 1) as underlined with grey colour

Calibration plots were assessed for nine propensity score models for which the full prediction rule was published. As visualized in Figure 1, two models showed acceptable calibration (Benhalima‐1 and Benhalima‐2 2020), two models overestimated (Göbl 2012 and Sweeting 2017) and six models underestimated the risk of GDM (van Leeuwen 2010, Nanda 2011, Savona‐Ventura 2013, Gabby‐Benziv 2015 and Syngelaki 2015) when calculated as originally published. The agreement between predicted vs. observed probabilities was notably improved after recalibration (Figure 2): Six out of nine models had acceptable to almost perfect calibration (Göbl 2012, Savona‐Ventura 2013, Gabby‐Benziv 2015, Sweeting 2017, Benhalima‐1 and Benhalima‐2 2020), whereas three models showed sporadic overestimation (van Leeuwen 2010, Nanda 2011, Syngelaki 2015).

### 3.4 Variable importance and net benefit analysis

**FIGURE 3:**
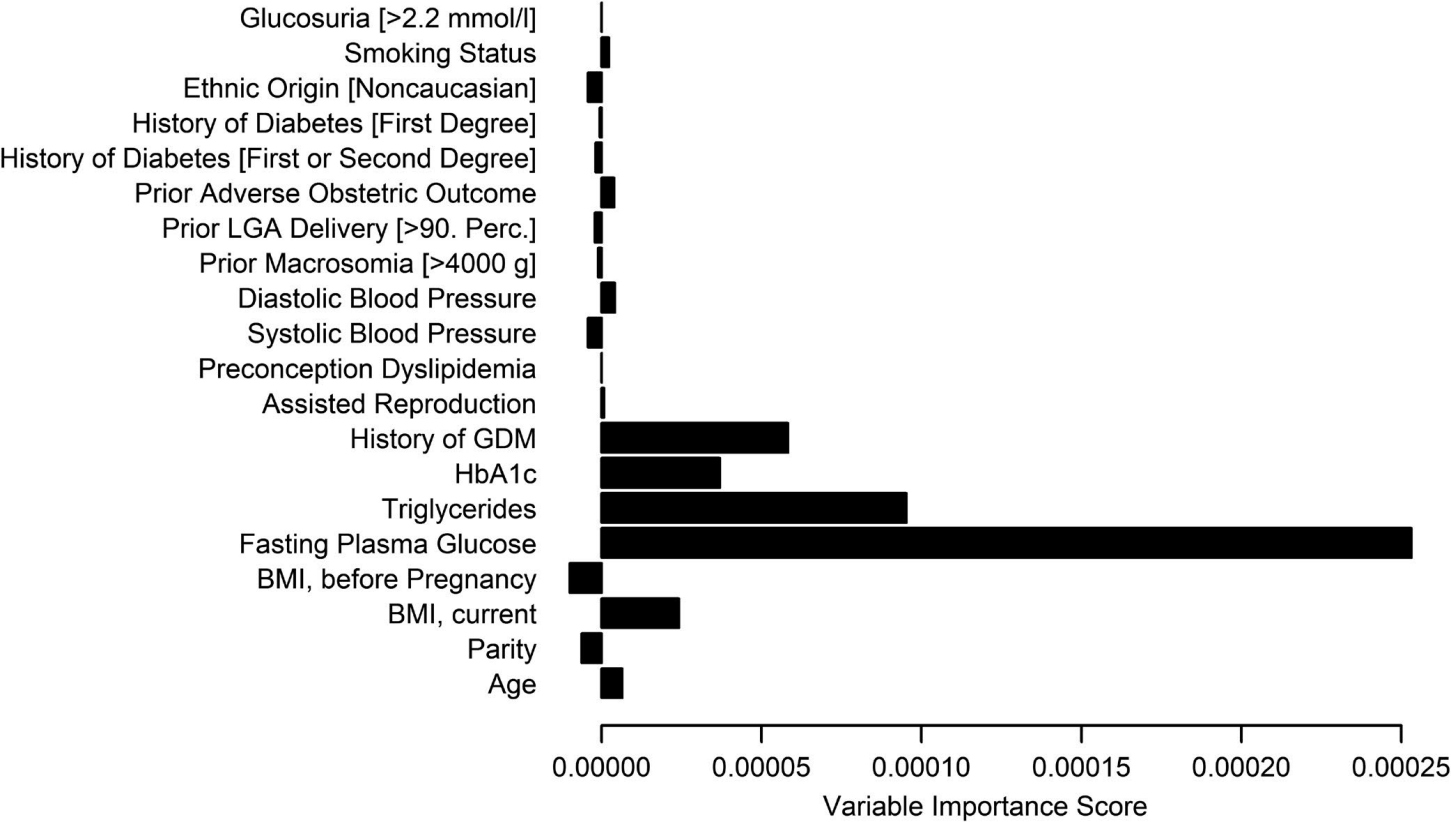
Variable importance scores assessed by random forest analysis

**FIGURE 4:**
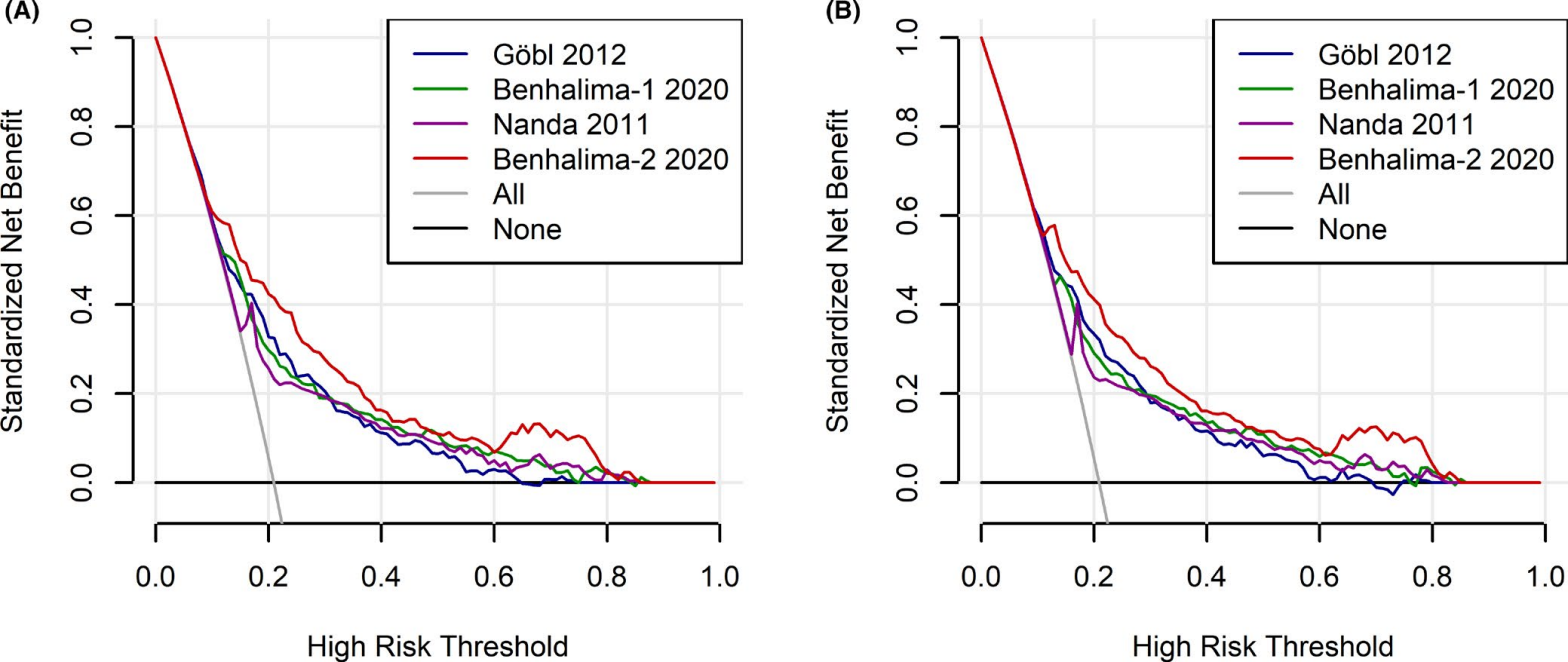
Decision curve analysis representing the net benefit (expressed as the net proportion of true positive cases). Decision curves for the four models with best discrimination are shown calculated according to the original publication (A) and after recalibration (B). The solid grey line represents the net benefit when all patients are treated as high risk patients who will develop GDM (All). The solid black line represents the net benefit when none of the patients are considered to develop GDM (None). A model with higher net benefit is preferred

Random forest analysis revealed highest variable importance scores for history of GDM in previous gestation as well as laboratory parameters like fasting plasma glucose, triglycerides and HbA1c (Figure 3). As a consequence, the Benhalima‐2 2020 model, which included these variables, showed superior net benefit (ie the net proportion of true positive cases) as compared to other models. The decision curves of the four models with best discrimination (that is the highest ROC‐AUC) are provided in Figure 4.

## 4 DISCUSSION

In this prospective validation study, we aimed to investigate the accuracy of low‐invasive prognostic models to predict the later development of GDM. A total of fifteen published algorithms were analysed which showed moderate to fair discrimination—this means that their ability to distinguish low from high risk individuals at the beginning of pregnancy was acceptable with areas under the ROC curve up to 76.9%. Although the performance was adequate in terms of discrimination, the agreement between predicted probability and observed risk (ie the calibration) of most models was poor. A poor calibrated risk estimate, however, is a non‐negligible limitation as it can lead to inaccurate risk predictions and false expectations.^31^ Calibration performance (the ‘Achilles heel of predictive analytics’) is markedly affected by the characteristics of the setting in which a model is developed, including the diagnostic criteria used to define the disease and consequently resulting number of affected cases (low disease incidence in the evaluation cohort can lead to an underestimated risk of the disease, and the opposite for high incidence).^31^ This is of particular importance for risk prediction in the field of GDM as the diagnostic algorithms were adapted over time affecting indices of the disease prevalence.^2^ Based on the results of the Hyperglycemia and Adverse Pregnancy Outcomes (HAPO) study,^32^ the IADPSG proposed novel criteria for the diagnosis of GDM with lower thresholds in 2010, which were later adopted by many health care organizations, including the WHO in 2013, and implemented in many countries.^1^, ^8^ Lowering the diagnostic thresholds, however, was associated with identifying more women with moderate hyperglycaemia (ie patients who were previously defined as NGT) and therefore with a higher prevalence of GDM. As a consequence, especially those risk assessment tools that were originally developed based on older criteria (eg van Leeuwen 2010, Nanda 2011, Savona‐Ventura 2013, Syngelaki 2015) showed lower disease incidence (between 2.4% and 8.7%)^5^, ^15^, ^16^, ^20^ and hence tended to underestimate the risk for GDM when applied in populations where the most recent IADPSG criteria were considered as is the case in our study. Notably, we observed good calibration for both models most recently proposed by Benhalima et al, who used the recent IADPSG definition to identify women with hyperglycaemia.^6^ In our study, two models overestimated the risk for developing GDM (Göbl 2012 and Sweeting 2017)^17^, ^18^ and, again, poor calibration can be explained by the respective study settings: The model proposed by Göbl et al was originally developed to estimate the risk for GDM in women with normal fasting glucose (< 92mg/dl). Moreover, that study was focussed on women with risk factors and therefore ended up with a markedly high incidence of hyperglycaemia in the evaluation cohort as addressed by the authors.^17^ The second study used early OGTTs at the first appointment in patients with risk factors (non‐Caucasian ethnicity, age ≥40 years as well as maternal overweight or obesity) which was repeated at 18 to 20 weeks and at 24 to 28 weeks if the previous test was still negative for GDM diagnosis.^18^ Repeated OGTT testing before 24 weeks in patients with risk factors is, however, not recommended by other guidelines. Finally, the agreement between observed and predicted risk for GDM was markedly improved by recalibration of the models. Only three recalibrated risk assessment tools showed sporadic overestimation (van Leeuwen 2010, Nanda 2011 and Syngelaki 2015).^5^, ^15^, ^16^

Two further studies systematically evaluated the accuracy of multiple prognostic models to predict the risk for GDM. Lamain‐de Ruiter et al assessed twelve risk assessment tools within a well‐designed external validation study from the Netherlands and observed ROC‐AUC statistics between 67% and 78%.^33^ In line with these results, another Dutch study published by Meertens and coworkers reported a discriminative performance of several prediction algorithms between 68% and 75%,^34^ which is comparable to our results. The agreement between observed and predicted risk of GDM was improved after recalibration in both studies. In particular, Meertens et al concluded that nearly all models overestimated the risk for GDM when the models were used as originally published (ie without recalibration). As both Dutch studies used a selective screening approach based on risk factors to identify women with GDM (meaning that only women with risk factors received a diagnostic OGTT where the more stringent WHO 1999 thresholds were applied), both validation cohorts ended up with a low disease incidence of 2.4% (127 affected cases) and 4.9% (181 affected cases), respectively.^33^, ^34^ As explained above, different diagnostic criteria and disease incidence in evaluation and validation cohorts could markedly influence model accuracy (especially calibration). This underlines the importance of our study wherein GDM was diagnosed by universal OGTT testing and glucose thresholds in accordance with the most recent guidelines.^1^, ^8^

Our study further extends previous research as we included a detailed examination of variable importance to identify the most prominent risk factors. We found highest variable importance scores for history of GDM in previous pregnancy, as well as elevated routine laboratory parameters, such as fasting plasma glucose, HbA1c and triglycerides. The prognostic value of fasting glucose and HbA1c at early gestation was assessed in previous studies, and discriminative values of approximately 65% for both parameters are reported in the literature.^35^, ^36^, ^37^ In contrast, the prognostic value of maternal lipids is less well investigated, although elevated maternal triglyceride concentrations were found to be associated with adverse pregnancy outcome.^38^ Moreover, Bao at al. recently found higher plasma triglycerides in women who developed GDM at early and mid‐gestation.^39^ It is noteworthy to mention that the prediction model that showed the best model accuracy (Benhalima‐2 2020) consisted of a combination of anamnestic risk markers in addition to routinely assessable biochemical variables.^6^ In addition, the net benefit of this model (defined as the net proportion of true positive cases) across a wide range of threshold probabilities was superior to other algorithms.

In Austria, like in other Central European countries, medical care for pregnant women is provided by gynaecologists and is further supported by general practitioners and midwifes. Patients who develop gestational diabetes are mostly transferred to qualified institutions. This underlines the relevance of clinical prediction models, which can help to identify subgroups with particularly high risk already at early gestation. Moreover, amidst the COVID‐19 pandemic we are confronted with challenge of upholding screening standards for GDM while limiting in‐person contact for the purpose of reducing the risk of exposure and dissemination of COVID‐19. The absence of an adequate screening strategy could result in increased numbers of undiagnosed and untreated GDM cases and consequently GDM related complications, particularly in countries that practice universal screening. Selective screening based on the presence of one or more risk factors has shown to have limited diagnostic accuracy.^4^ Under those circumstances, the opportunity arises to utilize risk prediction models in order to improve the existing selective screening algorithms and therefore to effectively reduce the number of inconvenient and unnecessary oral glucose tolerance tests.

Advantages and limitations need to be addressed: The prospective study design as well as the large number of included cases, and especially of women developing GDM, is a clear advantage of this work as risk factors with lower prevalence were captured accurately. Thereby, the number of affected cases is even higher as in other studies in this field.^33^, ^34^ Moreover, with our sample size, we were able to detect a prediction model with a ROC‐AUC of at least 57.4% as statistically significant. As the predictive accuracy (in terms of discrimination) of several investigated prediction models ranged between 60.7% and 76.9%, we conclude that our study had sufficient power. The single‐centre approach could be criticized. However, to our knowledge, this is the first validation study that used the most recent GDM diagnostic recommendations. The disease incidence is comparable with that observed in the HAPO study (ie 18% in the entire HAPO cohort, and between 9.3% and 25.5% in the study centres).^40^ Hence, there are no compelling arguments against the validity of our study and the generalizability of our results for Central European settings using the most recent diagnostic criteria. Although ethnic origin was examined in detail in our study, it has to be mentioned that the prevalence of risk factors may vary in different ethnicities and may possibly affect the predictive accuracy of prediction models. Therefore, we recommend further investigations in non‐European populations.

There are several concluding remarks: First, model accuracy, and especially calibration, could be influenced by the diagnostic criteria and disease incidence in either evaluation or validation cohorts. This is of particular importance in the field of GDM prediction as diagnostic criteria changed over time and varied between the studies. Second, a detailed assessment of variable importance underlined the additional relevance of routinely available biochemical variables such as fasting glucose, HbA1c and triglycerides, to further improve clinical prediction models. Third, the model recently proposed by Benhalima et al^6^ performed satisfactorily in terms of discrimination and calibration. It can be either used as a basis to develop future models with improved predictive accuracy or tested in clinical studies aiming to reduce the risk for GDM and adverse obstetric outcomes by timely interventions.

## CONFLICT OF INTEREST

The authors declare that they have no competing interests.

## AUTHOR CONTRIBUTIONS

CSG conceived the study. Data assessment and patient recruitment was performed by IR, GK, JB, DE and CSG. Calculations and data interpretation were performed by CSG, AT and MM. Statistical analysis was performed by CSG. CSG prepared tables and figures. The manuscript was written by CSG and GK. AT, GYS, CS, JB, IR, PH, DE, EAH and WE critically revised the manuscript. All authors reviewed and edited the final draft of the manuscript. The authors have nothing to disclose. CSG and GK serve as guarantors and accept full responsibility for the work and/or the conduct of the study, had access to the data and controlled the decision to publish.

## Supporting information

Supplementary Material
