# Supplementary material for: Performance of early risk assessment tools to predict the later development of gestational diabetes
Source: Eur J Clin Invest. 2021 Jun 18;51(12):e13630. doi: 10.1111/eci.13630 (PMC9285036; doi:10.1111/eci.13630)

**Supplementary Data 1**

**Table S1:** Mathematical expression of clinical prediction models analyzed in this study.

| **Author** | **Risk Score Calculation** | **Criteria** |
| --- | --- | --- |
|  |  |  |
| **Naylor 1997** (10) | *Sum score model:* sum (β × x) whereby β × x = 1 × age [31-34 years] + 2 × age [≥ 35 years] + 2 × pre-pregnancy BMI [22.1-25.0 kg/m²] + 3 × pre-pregnancy BMI [≥ 25.1 kg/m²]+ 5 × ethnic origin [Asian] + 2 × ethnic origin [others than white, black or Asian] | Carpenter & Coustan 1982 3h-100g OGTT  Pretest: 50g-GCT |
| **Caliskan 2004** (11) | *Sum score model:* sum (β × x) whereby x = 1 × age [≥ 25 years] + 1 × pre-pregnancy BMI [≥ 25 kg/m²] + 1 × prior adverse obstetric outcome[recurrent abortions (> 2 abortions), fetal anomaly despite normal karyotype, unexplained fetal death ≥ 20 weeks] + 1 family history of diabetes[first degree] + 1 × prior macrosomia [> 4000 g] | NDDG 1979 3h-100g OGTT Pretest: 50g GCT |
| **Shirazian 2009** (14) | *Sum score model:* sum (β × x) whereby β × x = 1 × age [25-29 years] + 3 × age [≥ 30 years] + 1 × pre-pregnancy BMI [25.0-29.9] + 2 × pre-pregnancy BMI [≥ 30.0]+ 1 × family history of diabetes [first degree] | Carpenter & Coustan 1982 75g OGTT Pretest: none |
| **Phaloprakarn 2009** (13) | *Sum score model:* sum (β × x) whereby β × x = 6 × age [years] + 11 × first trimester BMI [kg/m²] + 109 × family history of diabetes [first degree] + 42 × prior macrosomia [≥4000g] + 49 × history of ≥2 abortions | Carpenter & Coustan 1982 3h-100g OGTT Pretest: 50g GCT |
| **Teede 2011** (12) | *Sum score model:* sum (β × x) whereby β × x = 1 × age [25-34 years] + 2 × age [≥ 35 years] + 1 × first trimester BMI [20-34.9 kg/m²] + 2 × first trimester BMI [≥ 35 kg/m²]+ 1 × ethnic origin [Polynesian, Maritime Southeast Asian, East Asian, South Asian, African] + 2 × ethnic origin [Mainland Southeast Asian] + 1 × family history of diabetes [first degree] + 2 × history of GDM | ADIPS 1998 75g OGTT Pretest: 50g GCT |
| **Pintaudi 2014** (21) | *Decision tree model:* four classes (from low-risk to high risk) FPG [ ≤ 4.4 mmol/l] FPG [ > 4.4 ≤ 5.1 mmol/l] & pre-pregnancy BMI [ ≤ 24.4 kg/m²]  FPG [ > 4.4 ≤ 5.1 mmol/l] & pre-pregnancy BMI [ > 24.4 kg/m²] FPG [ > 5.1 mmol/l] | IADPSG 2010 75g OGTT Pretest: none |
| **van Leeuwen 2010** (5) | *Propensity score model:* exp(β × x)/[1+exp(β × x)] whereby β × x = −6.1 + 0.83 × ethnic origin [Non-Caucasian] + 0.57 × family history of diabetes [first or second degree] - 0.67 × multipara without history of GDM + 0.5 × multipara with history of GDM + 0.13 × pre-pregnancy BMI [kg/m²] | WHO 1999 75g OGTT Pretest: RG, GCT |
| **Nanda 2011** (15) | *Propensity score model:* exp(β × x)/[1+exp(β × x)] whereby β × x = – 8.68947 + 0.05365 × age [years] + 0.10852 × first trimester BMI [kg/m²] + 1.00312 × ethnic origin [South Asian] + 0.88785 × ethnic origin [East Asian] + 3.72259 × history of GDM + 0.67673 × prior macrosomia [>90th percentile] | WHO 1999 75g OGTT Pretest: RG, RF |
| **Göbl 2012** (17) | *Propensity score model:* exp(β × x)/[1+exp(β × x)] whereby β × x = −5.72 + 1.16 × history of GDM + 0.94 × glycosuria [>2.2 mmol/l] + 0.08 × age [years] + 0.46 × family history of diabetes [first or second degree] + 1.38 × preconception dyslipidaemia + 0.63 × ethnic origin [Asian, African, Hispanic, Indian] + 0.48 × FPG [mmol/l] | IADPSG 2010 75g OGTT Pretest: none |
| **Savona-Ventura 2013** (20) | *Propensity score model:* exp(β × x)/[1+exp(β × x)] whereby β × x = -4.144 + 3.142 × FPG [> 5.0 mmol/L] + 0.758 × age [≥ 30 years] + 0.543 × diastolic blood pressure [≥ 80 mmHg] | ADA 2003 75g OGTT Pretest: none |
| **Syngelaki 2015** (16) | *Propensity score model:* exp(β × x)/[1+exp(β × x)] whereby β × x = – 4.0050 + previous GDM × (3.9209 + 0.0206 × (weight – 69 kg)) + nulliparous or parous with no previous GDM × (– 0.7885 × parous without previous GDM + 0.0807 × (age – 35 years) + 0.0381 × (weight – 69 kg) – 0.0591 × (height – 164 cm) + 0.9332 × family history of diabetes [first degree] + 0.5869 × family history of diabetes [second degree] + 0.4712 × ovulation drugs + 0.4562 × ethnic origin [Afro-Caribbean] + 1.0727 × ethnic origin [East Asian] + 0.8401× ethnic origin [South Asian] + 0.2247 × birth weight of previous pregnancy [Z-score]) | WHO 1999 75g OGTT Pretest: RG, RF |
| **Gabbay-Benziv 2015** (19) | *Propensity score model:* exp(β × x)/[1+exp(β × x)] whereby β × x = – 11.569 + 0.064 × age [years] + 2.026 × ethnic origin [(Northeast) Asian] + 0.083 × ethnic origin [black] + 0.493 × ethnic origin [Hispanic] + 1.661 × ethnic origin [Other Non-white] + 2.144 x prior GDM + 0.034 x systolic blood pressure [mmHg] + 0.082 x first trimester BMI [kg/m²] | Carpenter & Coustan 1982 3h-100g OGTT Pretest: 50g GCT |
| **Sweeting 2017** (18) | *Propensity score model:* exp(β × x)/[1+exp(β × x)] whereby β × x = – 5.9515 + 2.8624 × history of GDM + 1.55 × ethnic origin [East Asian] + 1.7033 × ethnic origin [South Asian] + 3.0678 × family history of diabetes [first or second degree] – 0.5836 × parity + 0.0354 x age [years] + 0.08462 x first trimester BMI [kg/m²] | ADIPS 2014 (modified) 75g OGTT Pretest: RF, GCT |
| **Benhalima-1 2020** (6) | *Propensity score model:* exp(β × x)/[1+exp(β × x)] whereby β × x = – 1.39 + 0.41 × family history of diabetes [first degree] + 0.35 x history of smoking before pregnancy + 1.19 × ethnic origin [Asian] + 0.08 × age [years] – 3.40 × height [m] + 0.07 x first trimester BMI [kg/m²] + 2.05 x history of GDM | IADPSG 2010 75g OGTT Pretest: none |
| **Benhalima-2 2020** (6) | *Propensity score model:* exp(β × x)/[1+exp(β × x)] whereby β × x = -6.38 + 1.92 × history of GDM + 0.08 x FPG [mg/dl] – 5.56 x height [m] + 0.006 x Triglycerides [mg/dl] + 0.07 x age [years] + 0.56 x ethnic origin [Non-Caucasian origin] + 0.015 x first trimester weight [kg] + 0.42 x family history of diabetes [first degree] + 0.61 x HbA1c [%] | IADPSG 2010 75g OGTT Pretest: none |

Abbreviations are ROC-AUC, area under the receiver operating characteristic curve; FPG, fasting plasma glucose; BMI, body mass index; HbA1c, glycated hemoglobin A1c; GCT, glucose challenge test; RF, risk factors; RG, random glucose

**Table S2:** Discrimination of clinical risk prediction models. ROC-AUC statistics are presented for the original and the actual cohort as well as for singleton pregnancies and nulliparous women

| **Author** | **ROC AUC (%) Original Cohort** | **ROC AUC (%) Actual Cohort** | **ROC AUC (%) Actual Cohort Singleton Pregnancies** | **ROC AUC (%) Actual Cohort Nulliparous** |
| --- | --- | --- | --- | --- |
|  |  |  |  |  |
| **Naylor 1997** (10) | 69.0 | 65.5 | 65.2 | 63.6 |
| **Caliskan 2004** (11) | not reported | 64.5 | 63.4 | 62.3 |
| **Shirazian 2009** (14) | not reported | 60.7 | 60.2 | 56.7 |
| **Phaloprakarn 2009** (13) | 76.9 Internal validation: 75.2 | 67.6 | 67.0 | 66.6 |
| **Teede 2011** (12) | 70.3 | 68.9 | 69.1 | 63.5 |
| **Pintaudi 2014** (21) | not reported | 67.7 | 68.1 | 66.1 |
| **Van Leeuwen 2010** (5) | 77.0 | 70.8 | 71.6 | 66.8 |
| **Nanda 2011** (15) | 78.8 | 72.9 | 73.4 | 65.7 |
| **Göbl 2012** (17) | 71.0 Internal validation: 74.0 | 71.7 | 72.6 | 66.9 |
| **Savona-Ventura 2013** (20) | 87.1 | 65.2 | 65.3 | 65.4 |
| **Syngelaki 2015** (16) | 82.3 | 71.5 | 73.0 | 69.6 |
| **Gabbay-Benziv 2015** (19) | 81.9 | 71.6 | 72.1 | 65.3 |
| **Sweeting 2017** (18) | 88.0 | 71.2 | 71.4 | 68.2 |
| **Benhalima-1 2020** (6) | 72.0 After cross-validation: 68.0 | 71.7 | 72.2 | 66.5 |
| **Benhalima-2 2020** (6) | 76.0 After cross-validation: 72.0 | 76.9 | 77.7 | 73.9 |

**Figure S1:** Flow-chart representing included and excluded patients


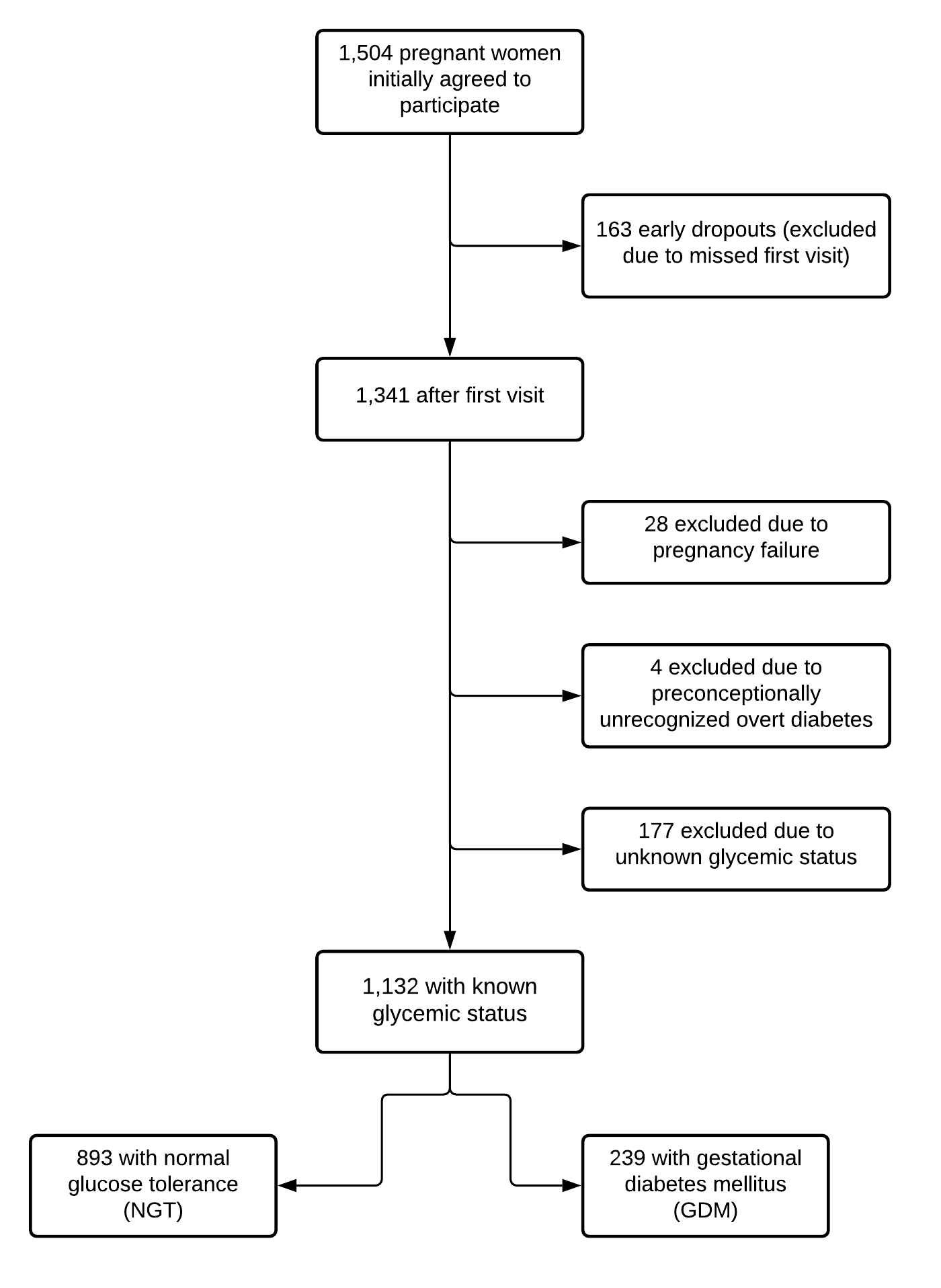

Supplement: Supplementary file 1 — Supplementary Material [file ECI-51-0-s001.docx]
